# Identification of host biomarkers from dried blood spots for monitoring treatment response in extrapulmonary tuberculosis

**DOI:** 10.1038/s41598-022-26823-6

**Published:** 2023-01-12

**Authors:** Shizza Khalid, Atiqa Ambreen, Aasia Khaliq, Hafeez Ullah, Manal Mustafa, Tehmina Mustafa

**Affiliations:** 1 Center for International Health, Department of Global Public Health and Primary Care, University of Bergen, P.O box 7804, 5020 Bergen, Norway; 2 Department of Microbiology, Gulab Devi Hospital, Lahore, Pakistan; 3 Institute of Molecular Biology and Biotechnology (IMBB), The University of Lahore, Lahore, Pakistan; 4 Department of Life Sciences, Lahore University of Management Sciences (LUMS), Lahore, Pakistan; 5 Leverify (Business Intelligence Officer at Leverify, Lahore, Pakistan; 6 Department of Thoracic Medicine, Haukeland University Hospital, Bergen, Norway

**Keywords:** Biomarkers, Diseases

## Abstract

There is a lack of objective tools for monitoring treatment response in extrapulmonary tuberculosis (EPTB). This study aimed to explore the utility of inflammatory biomarkers from the dry blood spots (DBS) as a tool for monitoring treatment response in EPTB. In a prospective cohort study, 40 inflammatory biomarkers were investigated in DBS samples from 105 EPTB cases using a Luminex platform. The samples were taken before, and, at the end of the 2nd and 6th months of treatment. A total of 11 inflammatory host biomarkers changed significantly with treatment in all EPTB patients. CXCL9/MIG, CCL20, CCL23, CXCL10/IP-10, CXCL1, CXCL2, and CXCL8 significantly declined in our cohort of EPTB (48 TB pleuritis and 57 TB lymphadenitis) patients at both time points. A biosignature consisting of MIG, CCL23, and CXCL2, corresponded with the treatment response in 81% of patients in the 2nd month and 79% of patients at the end of treatment. MIG, CCL23, IP-10, and CXCL2 changed significantly with treatment in all patients including those showing partial clinical response at the 2nd month of treatment. The changes in the levels of inflammatory biomarkers in the DBS correspond with the treatment success and can be developed as a routine test in low-resource settings.

## Introduction

Tuberculosis (TB) is one of the top ten causes of death worldwide, ranking above HIV and AIDS. According to the WHO global figures, 16% of all TB incident cases are of extrapulmonary TB (EPTB). EPTB is a paucibacillary disease that can infect any anatomical site though lymph nodes and pleura are the most commonly infected organs^1–4^. All available tests for microbiological confirmation of the disease have limited sensitivity^5^, due to which EPTB is often treated empirically^6–8^. Treatment monitoring is crucial for assessing treatment response, which can ultimately result in reducing mortality and morbidity^9^. Currently, there is a lack of objective tools for monitoring treatment as all existing tools have a limited role^10,11^. An objective, robust, rapid, and inexpensive method, is necessary to gauge treatment response^12^. Host biomarkers produced during pathogen infection have the potential to be used as a surrogate tool for therapy monitoring^6,13,14^. Whole blood and plasma sampling are often impractical for treatment monitoring especially in low-resource, TB-endemic areas^15,16^. Dried blood spots (DBS), on the other hand, offer a simple, robust, cost-effective method of sampling, with fewer logistical requirements and higher patient accessibility^16–18^. This study is part of a larger project, in which similar 40 inflammatory biomarkers were investigated in unstimulated plasma for monitoring treatment response in EPTB^19^. In this study, we intend to investigate the same set of biomarkers in DBS as markers for treatment response in EPTB. We wanted to observe how many of these biomarkers change significantly with treatment and their potential to be developed as a routine test.

## Results

### Patients characteristics

**Figure 1:**
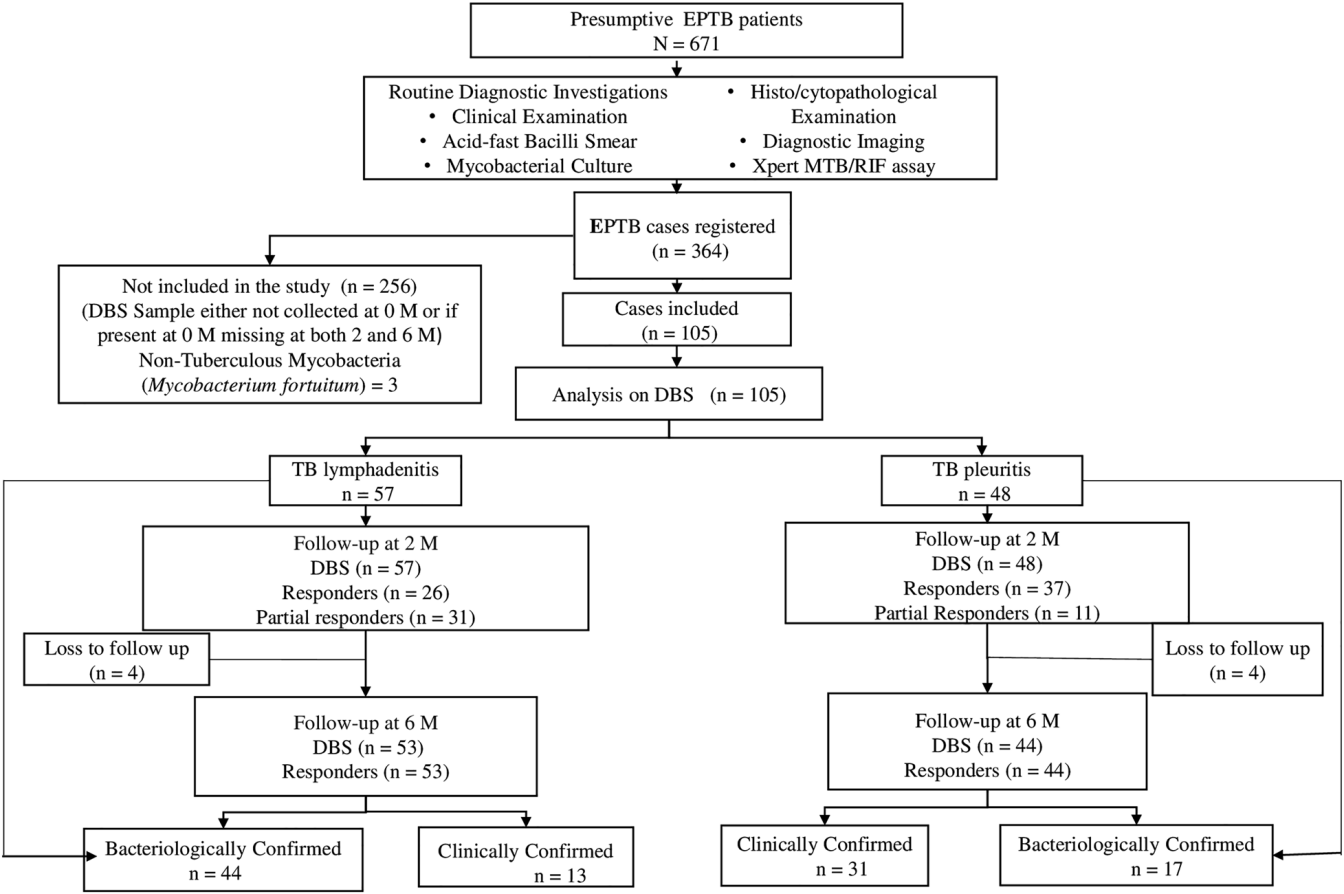
A flow chart showing the study design and the timeline of DBS collection during the study. *DBS* dried blood spots, *EPTB* extrapulmonary tuberculosis, *M* month of the treatment, *N* total number, *n* number in subgroups.

Of the total 364 EPTB patients registered, 105 patients were included in the study and were followed prospectively during the course of treatment. The remaining patients were non-compliant and unavailable for providing samples. Figure 1 shows the inclusion criteria and classification based on the type and bacteriological status of the cases. The cohort comprised of mainly two types of EPTB patients, 57 TB lymphadenitis, and 48 TB pleuritis cases. A total of 61 patients were bacteriologically confirmed, while the remaining were clinically diagnosed based on the composite reference standard. All patients received anti-TB treatment according to the WHO standard guidelines for drug-susceptible TB^20^. During their treatment, blood samples were collected at 2nd and 6th months. At the end of the two months, 45% of TB lymphadenitis and 77% of TB pleuritis cases responded to the treatment. However, the percentage of responders rose to 68% amongst TB lymphadenitis and 89% amongst TB pleuritis patients, by the end of 6 months. Almost half of the patients that responded partially to the treatment after two months ultimately responded at the end of six months. All cases showed clinical improvement at the end of their treatment duration. A total of 8 patients were lost to follow-up after two months of treatment.

As seen in Table 1, the cohort had a higher percentage of females amongst the TB lymphadenitis patients. A male predominance was seen amongst the TB pleuritis cases with a median age of 20 and 25 years, respectively. Only one TB pleuritis patient had a random blood glucose level of more than 200 mg/dl at the time of inclusion in the study.

**Table 1:** Demographic and clinical characteristics of extrapulmonary tuberculosis patients.

Patient characteristics	TB lymphadenitis N = 57	TB pleuritis N = 48	p-value
Age in years, median, (range)	20 (11 – 72)	25 (15 – 70)	0.143
**Sex, n (%)**			
Male	16 (28)	35 (72)	< 0.001
Female	42 (72)	13 (28)	
**HIV status, n (%)**			
Positive	0 (0)	0 (0)	
Negative	57 (100)	48 (100)	
**History of diabetes, n/N (%)**			
Yes	0/57 (0)	1/48 (2)	0.407
No	53/57 (93)	45/48 (94)	
NA	4/51 (7)	2/48 (4)	
**Patient categorization, n/N (%)**			
Confirmed TB	44/57 (77)	17/48 (35)	< 0.001
Probable TB	13/57 (23)	31/48 (65)	
**Clinical response at 2 M of treatment, n/N (%)**			
Responders	26/57 (46)	37/48 (77)	0.011
Partial responders	31/57 (54)	11/48 (23)	
Clinical response at 6 M/end* of treatment, n/N (%)			
Responders	53/53 (100)	44/44 (100)	

*N* total number, *n* number, %: percentage, *NA* no information available, *TB* tuberculosis, *M* month.

*Treatment was extended for 21 patients beyond 6 months. Chi-square test was used to compare groups on categorical data. A p-value < 0.05 was considered significant.

### Significantly changed inflammatory biomarkers with treatment in all EPTB patients

**Figure 2:**
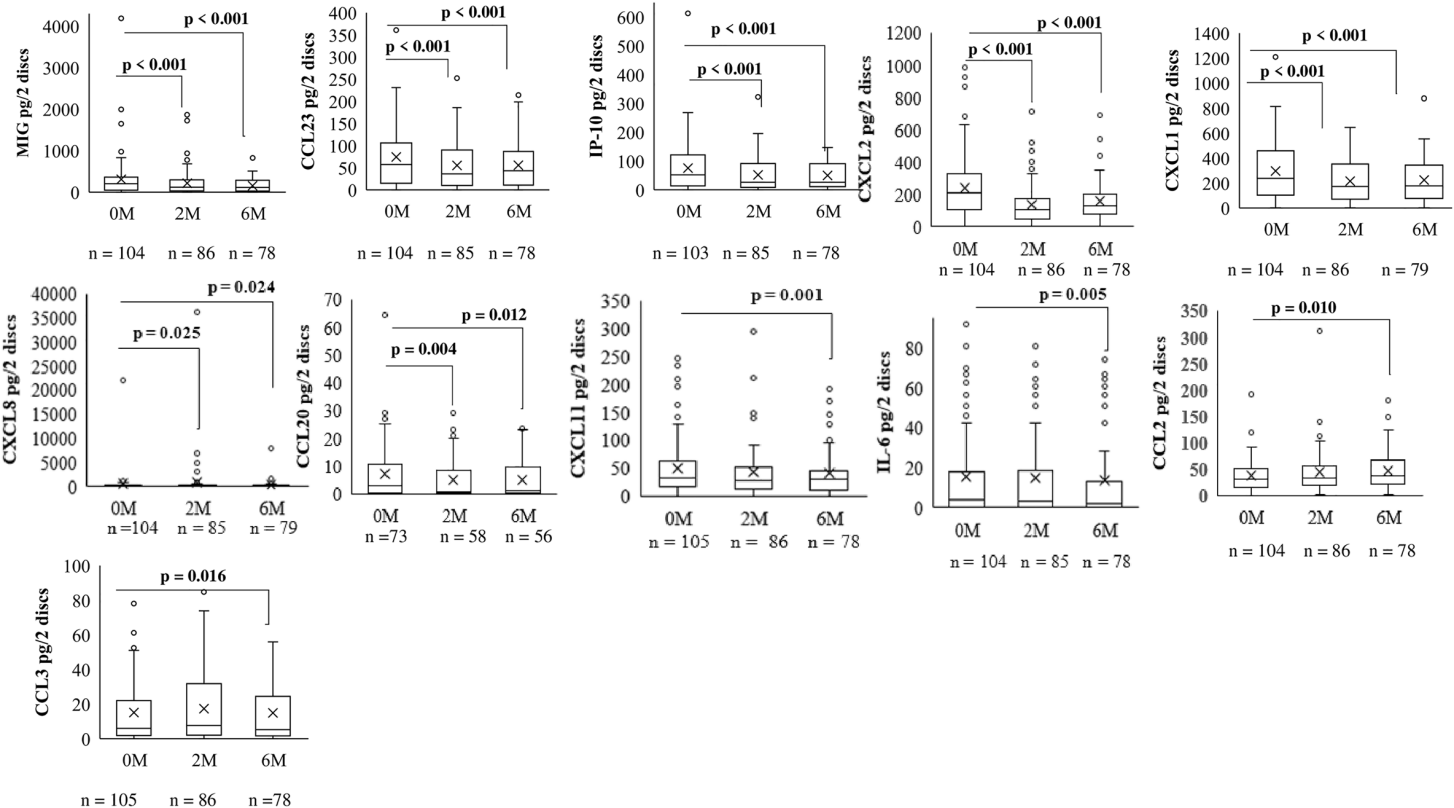
Box plots showing levels of inflammatory biomarkers in dried blood spots in all EPTB patients at baseline, 2nd, and 6th months of treatment. Only significantly changing biomarkers are shown. A p-value ≤ 0.25 was considered significant. Wilcoxon signed rank test was used for paired values. The boxes represent the median and interquartile range, while the whisker represents the minimum and maximum values. Different time points of treatment are shown on x-axis. *n* number of patients, *M* month of treatment.

All biomarkers were detected in elutes from DBS, and the following 11 biomarkers changed significantly in EPTB patients as shown in Fig. 2. The biomarkers that declined significantly at both time points during treatment as compared to the baseline were MIG/CXCL9 (2 M, p < 0.001, 6 M, p < 0.001), CCL23 (2 M, p < 0.001, 6 M, p < 0.001), IP-10/CXCL10(2 M, p < 0.001, 6 M, p < 0.001), CXCL1(2 M, p < 0.001, 6 M, p < 0.001), CXCL2 (2 M, p < 0.001, 6 M, p < 0.001), CXCL8 (2 M, p = 0.025, 6 M p = 0.024), CCL2 (2 M, p = 0.008, 6 M p = 0.012), and CCL20 (2 M, p = 0.004, 6 M p = 0.012). Levels of CXCL11 (p = 0.001), IL-6 (p = 0.005), CCL2 (p = 0.010), and CCL3 (p = 0.016), declined significantly only after 6 months as compared to the baseline levels.

### Significantly changed inflammatory biomarkers with treatment in TB Pleuritis patients

**Figure 3:**
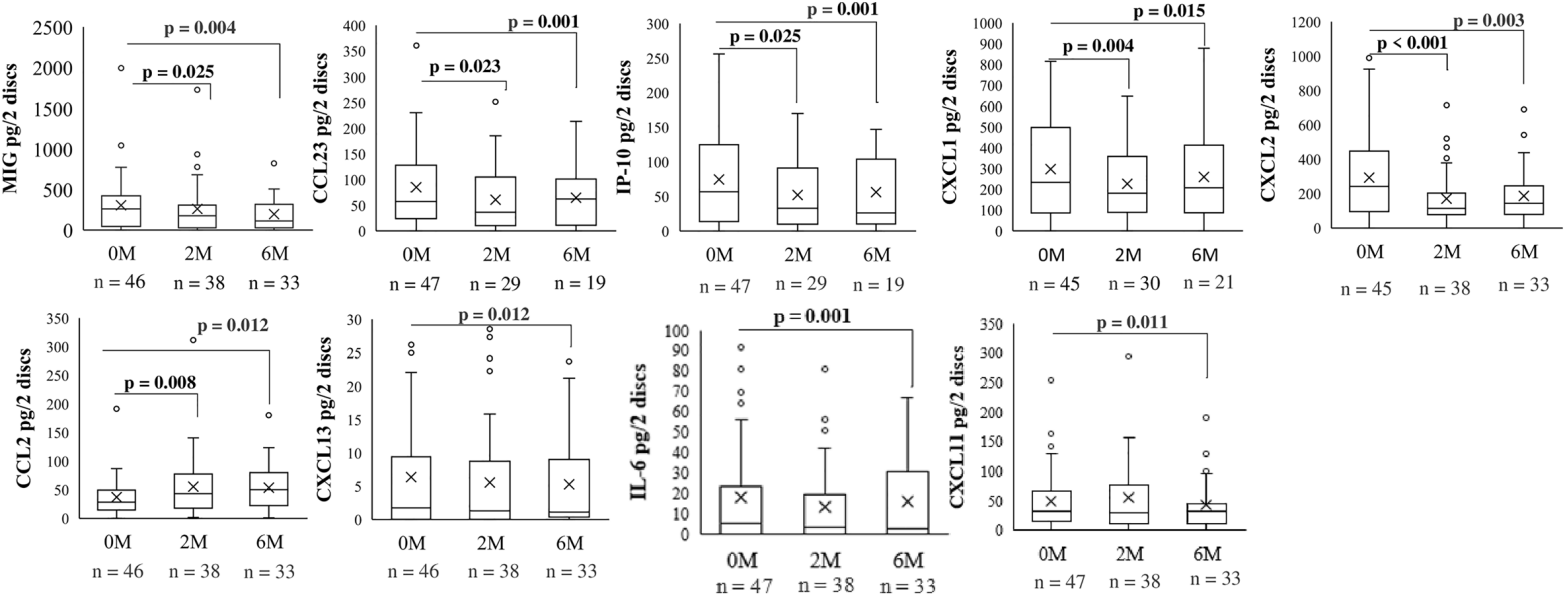
Box plots showing levels of inflammatory biomarkers in dried blood spots in Tuberculous (TB) pleuritis patients at baseline, 2nd, and 6th months of treatment. Only significantly changing biomarkers are shown. A p-value ≤ 0.25 was considered significant. Wilcoxon signed rank test was used for paired values. The boxes represent the median and interquartile range, while the whisker represents the minimum and maximum values. Different time points of treatment are shown on x-axis. *n* number of patients, *M* month of treatment.

A total of 9 inflammatory biomarkers changed significantly in TB pleuritis patients as shown in Fig. 3. The biomarkers that declined significantly at both time points during treatment as compared to the baseline were CXCL9/MIG (2 M, p = 0.025, 6 M, p = 0.004), CCL23 (2 M, p = 0.023, 6 M, p = 0.001), IP-10 (2 M, p = 0.025, 6 M, p = 0.001), CXCL1 (2 M, p = 0.004, 6 M p = 0.015), and CXCL2 (2 M, p < 0.001, 6 M, p = 0.003). Levels of CCL2 (2 M, p = 0.008, 6 M p = 0.012) increased at 2 and 6 months as compared to the baseline levels. Whereas, levels of IL-6 (p = 0.001), CXC11 (p = 0.011), and CXCL13 (p = 0.012) declined significantly only at the end of the 6 months as compared to the baseline levels. The biomarkers that did not show significant change with treatment in TB pleuritis patients are shown in Supplementary Fig. S1.

### Significantly changed inflammatory biomarkers with treatment in TB lymphadenitis patients

**Figure 4:**
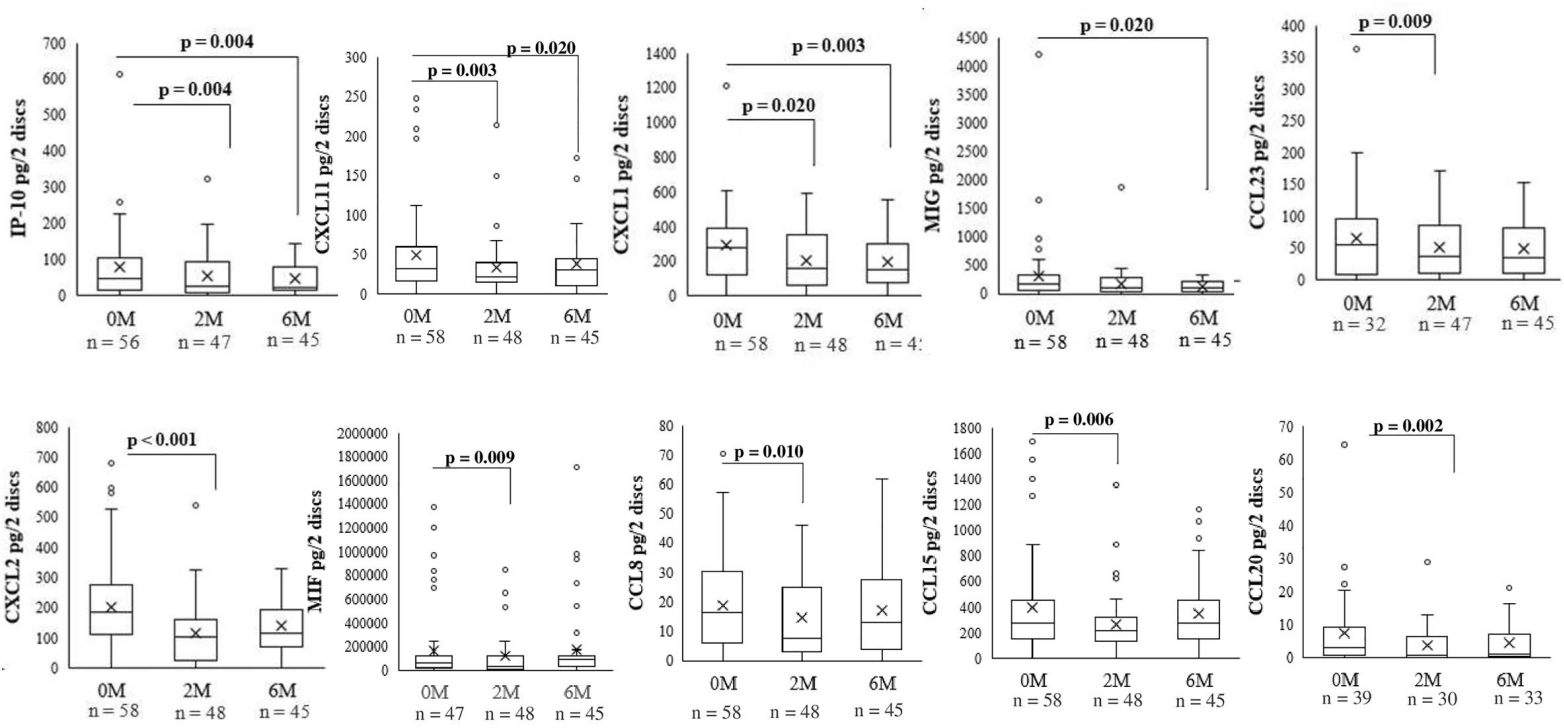
Box plots showing levels of inflammatory biomarkers in dried blood spots amongst the Tuberculous (TB) lymphadenitis patients at baseline, 2nd, and 6th months of treatment. Only significantly changing biomarkers are shown. A p-value ≤ 0.25was considered significant. Wilcoxon signed rank test was used for paired values. The boxes represent the median and interquartile range, while the whisker represents the minimum and maximum values. Different time points of treatment are shown on x-axis. *n* number of patients, *M* months of treatment.

In TB lymphadenitis patients, 10 biomarkers changed significantly with treatment as shown in Fig. 4. Levels of IP-10 (2 M p = 0.004, 6 M p = 0.004), CXCL1 (2 M, p = 0.020, 6 M, p = 0.003), and CXCL11 (2 M, p = 0.003, 6 M p = 0.020) declined at the second and the sixth months after treatment as compared to the baseline. While levels of CCL23 (p = 0.009), CXCL2 (p < 0.001), CCL20 (p = 0.002), CCL15 (p = 0.006), CCL8 (p = 0.010), and MIF (p = 0.009) changed significantly only at the second month of treatment. Levels of CXCL9/MIG (p = 0.020) declined significantly only at 6 months of treatment. The biomarkers that did not show significant change with treatment in TB lymphadenitis patients are shown in Supplementary Fig. S2.

### Common inflammatory biomarkers that changed significantly in both TB pleuritis and TB lymphadenitis patients

**Figure 5:**
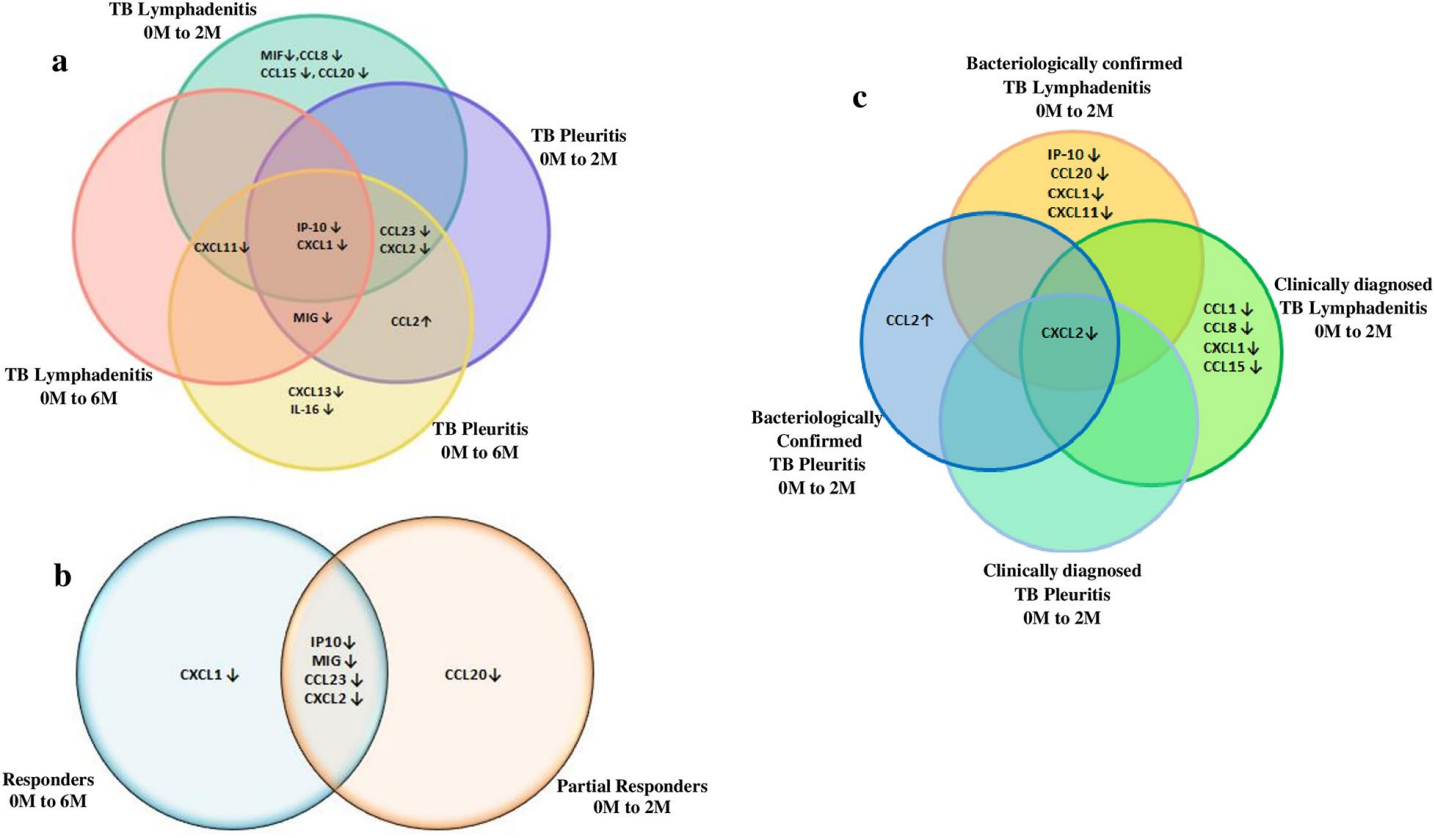
Common inflammatory biomarkers changing significantly with treatment amongst the Tuberculous (TB) lymphadenitis and TB pleuritis patients. (**a**) At the 2nd and 6th months of treatment compared to the baseline values among all TB lymphadenitis and TB pleuritis patients. (**b**) At the 2nd month of treatment compared to the baseline values among the responders and partial responders. (**c**) At the 2nd month of treatment compared to the baseline values among the bacteriologically confirmed and clinically confirmed cases. 0 M to 2 M: biomarkers changing significantly at 2 months as compared to the baseline:0 M to 6 M: biomarkers changing significantly at 6 months as compared to the baseline, ↓: show a decline in the level of biomarker and ↑: show an increase in the level of biomarker.

When biomarkers that changed independently in TB pleuritis and TB lymphadenitis were compared, only IP-10 and CXCL1 changed significantly at both time points in both groups of patients. Figure 5a shows different biomarkers changing at different time points in TB pleuritis and TB lymphadenitis patients.                                      

### Comparison of changes in biomarkers according to response to treatment

Based on the clinical response to treatment of the patients at 2 months, the patients were classified into responders and partial responders. Figure 5b, shows that MIG, CCL23, IP-10, and CXCL2 changed significantly with treatment at 2nd month in both groups.

### Comparison of biomarkers among bacteriologically confirmed and clinically confirmed cases

Figure 5c shows that there were differences in the profile of biomarkers changing with treatment between the bacteriologically confirmed and clinically diagnosed cases. Only CXCL2 significantly changed in both bacteriologically confirmed and clinically diagnosed cases of TB lymphadenitis and TB pleuritis patients.

### Magnitude and proportion of inflammatory biomarkers changes with treatment

A total of 13 biomarkers changed significantly in both types of EPTB. These biomarkers were not observed to change in all the patients. Table 2 shows the proportion of patients showing a ≥ 20% change in their levels of biomarkers at the 2nd and 6th months of treatment as compared to the baseline levels. The magnitude of change varied amongst the biomarkers, which was depicted by how many fold increase or decrease the biomarkers underwent when compared to their levels at the baseline. Overall, the range of change was from 2 to 13 folds. Amongst the TB lymphadenitis patients, CCL20 and MIF underwent a maximum fold change of 13-folds and 10-folds respectively after two months of treatment. Amongst the TB pleuritis patients, IL-6 and CXCL13 had the highest fold decline as compared to the baseline levels. IL-6 underwent a fivefold and a sixfold decline in its levels at the end of the 2nd and 6th months of treatment respectively when compared to the baseline.

**Table 2:** Proportion of tuberculous (TB) pleuritis and TB lymphadenitis showing a change of  > 20% in the levels of biomarkers in response to treatment and the magnitude of fold increase or decrease in the median levels. The biomarkers were detected from the dried blood spots.

Biomarkers	TB lymphadenitis				TB pleuritis			
	0–2 months		0–6 months		0–2 months		0–6 months	
	n/N (%)	Median folds ↓/↑	n/N (%)	Median folds ↓/↑	n/N (%)	Median folds ↓/↑	n/N (%)	Median folds ↓/↑
MIG	25/48 (52)	3↓	28/45 (62)	4↓	22/37 (59)	2↓	21/32 (65)	6↓
CCL23	42/57 (74)	3↓	36/57 (63)	3↓	18/38 (47)	3↓	23/33 (73)	3↓
IP-10	25/45 (56)	6↓	29/43 (67)	2↓	20/38 (53)	3↓	21/33 (64)	3↓
CXCL1	17/48 (35)	3↓	27/45 (60)	3↓	9/37 (24)	2↓	22/32 (69)	3↓
CCL20	18/29 (62)	13↓	18/35 (51)	5↓	16/28 (57)	3↓	15/23 (62)	5↓
CXCL2	33/48 (69)	5↓	27/45 (60)	2↓	27/37 (73)	2↓	22/33 (67)	2↓
MIF	14/46 (30)	10↓	12/45 (27)	5↑				
CXCL11	12/48 (25)	3↓	26/45 (58)	3↓	7/38 (18)	3↓	21/33 (64)	3↓
CCL8	27/48 (56)	3↓	23/45 (56)	6↓				
CCL15	25/48 (52)	2↓	22/45 (49)	2↓				
CCL2					22/37 (59)	2↑	21/32 (66)	2↑
IL-6					8/38 (21)	5↓	31/38 (81)	6↓
CCL3					12/38 (31)	2↑	16/33 (48)	2↓
CXCL13					11/37 (30)	4↓	22/32 (67)	8↓

*N* total number of patients, *n* number of patients showing > 20% change, Folds **↓/↑**: fold decrease or increase.

### A biosignature predicting response to treatment in the majority of patients

Levels of inflammatory biomarkers that declined consistently with treatment were selected to formulate a biosignature, for predicting treatment response in the maximum number of patients by the least possible number of biomarkers at both time points, and in both types of EPTB. As shown in Table 3, we narrowed down our selection to CXCL2, MIG, IP-10, and CCL23 as these were in an easily detectable range based on their median levels in DBS and showed large differences amongst the low and high-value change during treatment. As seen in Table 2, the fold change of these biomarkers ranged from 2 to 6 folds. A ≥ 20% decrease in any member of our proposed biosignature (MIG, CCL23, and CXCL2) detected 81% patients at the 2nd month and 79% patients at 6th month showing satisfactory response to treatment.

**Table 3:** Proportion of patients showing a significant change with treatment in the levels of four biomarkers individually and in combination constituting the biosignature, at 2nd and 6th month of treatment.

Immune biomarkers (Median at 0–2–6 M, pg/2 discs)	All samples (0 M–2 M) N = 86 n (%)	All samples (0 M–6 M) N = 78 n (%)	Responders (0 M–2 M) N = 52 n (%)	Partial responders (0 M–2 M) N = 33 n (%)
CXCL2 (209–107–132)	60 (70)	49 (63)	37 (71)	22 (67)
CCL23 (57–36–43)	49 (60)	47 (60)	26 (50)	22 (67)
MIG (203–120–113)	47 (55)	49 (63)	26 (50)	20 (61)
IP-10 (52–26–26)	45 (52)	51 (65)	26 (50)	18 (54)
MIG + CXCL2	66 (77)	60 (78)	40 (77)	25 (76)
IP-10 + CXCL2	66 (77)	61 (78)	40 (77)	25 (76)
CCL23 + CXCL2	69 (80)	58 (74)	41(79)	27 (81)
MIG + IP-10 + CXCL2	69 (80)	62 (79)	41 (79)	26 (79)
MIG + CCL23 + CXCL2	70 (81)	62 (79)	41 (79)	27 (81)

*N* total number of patients, *M* month of treatment, *n* number of patients showing ≥ 20% change. Python And Data Analysis was used to compute the various possible combinations. A combination consisting of least number of biomarkers and covering the maximum number of patients was selected. To formulate a biosignature, those patient samples were chosen that showed ≥ 20% change from the baseline levels.

## Discussion

This prospective cohort study was designed to test the hypothesis of whether it is possible to identify host biomarkers from the DBS for monitoring treatment efficacy in EPTB. All the candidate biomarkers have a well-established role in the pathogenesis of TB and were detected in the DBS. This study shows that levels of several biomarkers change significantly with successful TB treatment in EPTB patients. In order to identify the biomarkers that changed in majority of our patients, multiple biosignatures were extrapolated by selecting biomarkers that changed significantly with treatment at both time points in all EPTB patients. A biosignature of three biomarkers namely, MIG, CCL23, and CXCL2 was deduced to be a good candidate for indicating a good response to treatment. Changes in any of biomarkers included in this biosignature could predict response in 81% of patients after 2 months of treatment and in 79% of patients at the end of the treatment. Studies have shown that the use of a combination of biomarkers for monitoring a disease process is more beneficial^19,21^, especially in a disease like EPTB whose pathogenesis involves the interplay of several pro and anti-inflammatory cytokines and chemokines^22,23^.

Even though all the biomarkers showed a change in their levels during treatment, not all changed significantly. A few prerequisites were taken under consideration while selecting a biosignature. Firstly, biomarkers that showed a persistent decline with treatment were selected. Secondly, they had high baseline levels and showed a high fold change with treatment in the maximum number of patients. A high baseline level ensures that the biomarker of interest is easily detectable. These features could facilitate the future development of a more robust ELISA test that is low-cost and sustainable in routine clinical settings.

The cohort was classified into good responders and partial responders depending upon their clinical response to treatment at the 2nd month. The partial responders were taken as a proxy control group. MIG, CCL23, IP-10, and CXCL2 declined significantly at the 2nd month of treatment amongst both groups. Thus, a significant change in the biosignature comprising MIG, CCL23, and CXCL2 would also be able to predict response to treatment among those patients who lag in their clinical improvement at the end of the intensive phase of treatment at 2nd month and would assist in the correct management of patients.

The role of inflammatory biomarkers as candidates for treatment monitoring has been explored in some previous studies. A recent meta-analysis investigating the biomarkers that correlated with treatment response in active PTB by calculating fold change in unstimulated plasma levels identified CRP, IL-6, IP-10, and TNF-α as important in monitoring TB treatment and recommended that these should be explored in future studies^24^. Our 40-plex panel included 3 of these biomarkers (IL-6, IP-10, TNF-α). IP-10 and IL-6 levels also decreased significantly in our cohort of EPTB patients. There is also evidence of the decline in plasma levels of MIG, IP-10, GM-CSF, and IFN-γ corresponding with successful TB treatment, whereas levels of CCL2 and CCL11 increased with successful TB treatment in PTB patients^25^. We also demonstrated significant decrease in MIG and IP-10 levels and significant increase in CCL2 levels with successful TB treatment. The role of CFP-10, ESAT-6, and matrix metalloproteinases has also been investigated in the management of EPTB^26–28^. So far, IP-10 is the most promising and most studied biomarker for treatment monitoring of EPTB, in plasma and DBS^6,25,29^. Several studies are supporting its role as a potential surrogate marker for the management of EPTB, in both high and low-endemic countries. We also demonstrated that IP-10 levels from DBS samples changed significantly in our cohort of EPTB patients at both time points. However, IP-10 levels changed only in a proportion of EPTB patients, prompting the need to identify a combination of biomarkers that would predict response to treatment in a majority of patients. We decided to study the host biomarkers in DBS, as the use of plasma has its obvious set of limitations. Plasma sampling and transport cannot be done without a proper collection facility, trained staff, and infrastructure^15,17,18,30^. The storage and handling of biological proteins is quite crucial for the proteins to remain stable in plasma samples. The presence of anticoagulants, duration of storage, and dilutions make levels of biological proteins vulnerable to change. It is seen that many proteins are degraded during the storage and thawing processes, despite the maintenance of cold storage^30^. In comparison, proteins are stable in the DBS and easy to transport at ambient temperature and can be reliably eluted for the analysis of biomarkers. Due to these key features, DBS can be a simple, robust, non-invasive, and inexpensive method of sampling that does not require a cold chain to be maintained for its storage or transportation^6,17,18^. A finger prick of blood spot on a filter paper is all the sample needed^18^. This can be a very suitable sampling method for the delivery of good EPTB care by monitoring treatment response, especially in the low resource constraint areas of the world, that bear the maximum burden of disease^15,17,31^.

We previously reported utility of host inflammatory biomarkers in monitoring therapeutic response from unstimulated plasma of EPTB patients^19^. In this study we explored the possibility of using DBS to monitor treatment response in EPTB patients. Although there were many common biomarkers that changed with treatment, the biomarkers from DBS did not exactly match biomarkers from unstimulated plasma. Several external and intrinsic factors are known to affect the recovery of biomarkers from DBS. In contrast to plasma, a blood spot requires 50 micro liters of blood and this may affect the number of biomarkers recovered from DBS as compared to plasma. Secondly, lysis of certain cellular components occurs when blood spots are applied to the filter paper which may alter the levels of biomarkers in elutes prepared from the dried blood spots^32^. There is growing evidence that presence of red blood cells in the DBS samples may alter the final concentration of biomarkers in elutes^33–35^.

This study has some limitations. Firstly, there is no control group of non-responders. During the development of the study, it was assumed that some of the patients being treated empirically would not respond to treatment. However, all the patients responded, which could be due to the Hawthorne effect, leading to change in the practices of empirical treatment, and clinicians selecting patients more cautiously.

Another limitation of this study is the use of venous blood instead of capillary blood for preparing the blood spots. In routine, capillary blood is used for making blood spots via a finger prick. However, venous blood was used as this project was part of a larger study that involved investigating the levels of biomarkers in unstimulated plasma^19^. Numerous physiological differences exist between the capillary and the venous blood samples. Firstly, the concentration of hematocrit, hemoglobin, and cells like erythrocytes, and thrombocytes are found to be higher in capillary blood as compared to venous blood. The presence of certain viruses in the host may also affect the concentration of biomarkers based on the sample type. Biomarkers in lower concentration are more susceptible to the above-mentioned physiological differences^17^. Hence, if the same study is performed by using capillary blood for making DBS, the results may vary.

In summary, a biosignature of MIG, CCL23, and CXCL2, has a reliable predictive value in the treatment monitoring of the EPTB, at the second and sixth month of treatment, by using DBS. Validation studies are needed in different epidemiological settings with inclusion of a large number of EPTB patients. Once validated the findings have the potential to be developed into a robust ELISA-based test that can be used sustainably in low-resource settings.

## Methods

This study was carried out at Gulab Devi Hospital, Lahore, Pakistan, which is a semi-private tertiary care hospital that specializes in TB care^19^. Suspected TB patients are referred to the hospital from within the city and by the basic and district health units of the surrounding areas for further investigations and confirmation of diagnosis. Patients of all ages with presumptive EPTB, with no previous history of TB treatment (new TB cases) attending outpatient clinics, were enrolled from April 2016 to August 2017. Patients diagnosed as EPTB cases were registered and given standard anti-TB treatment. Regular follow-ups were done till a treatment outcome was documented. Written informed consent was taken from all the participants before the start of the study. Blood samples (5 ml) were collected from the patients at baseline (before the start of the treatment) and at the end of the second and/or sixth months of treatment. DBS were prepared by immediately placing one drop of collected blood on a properly labeled Whatman 903 protein saver card. The rest of the collected blood was transferred without delay into a vacutainer tube containing EDTA to avoid clotting. Care was taken to make an even distribution of blood spots in the designated area on the Whatman paper. After that, each paper was placed on a drying rack and allowed to dry for 3–4 hours at room temperature. Once dried, the filter papers were put in double-sealed bags with a desiccant bag and stored at minus 20 degrees Celsius until further use.

### Laboratory procedures

For TB lymphadenitis patients, affected lymph nodes were collected by excision biopsy, and for TB pleuritis patients at least 50–60 ml of pleural fluid was aspirated. Histological examination was performed on the biopsy samples and cytology on the pleural fluid smear. All samples were processed for smear for acid-fast bacilli, Xpert MTB/RIF assay (Xpert), and mycobacterial culture^36^. The smear was visualized with both Ziehl–Neelsen stain and Auramine O-stained smears under a Light-emitting diode Fluorescence microscope. Xpert was performed according to the manufacturer’s protocol^37^. For culture, two slopes of solid Lowenstein Jensen media and one mycobacterial growth indicator tube (MGIT™ 960™; Becton Dickinson, Sparks, MD, USA) were inoculated. The bacteriological confirmation was done either by culture or/and by Xpert in conjunction with smear microscopy.

### Selection of the inflammatory biomarkers for multiplex analysis

Biorad 40 plex Bio-Plex Pro™Human Chemokine Panel, was used on Luminex® xMAP™ to detect biomarkers from elutes of DBS. (Supplementary Table S1). The biomarkers fell under four groups. (i) Pro-inflammatory cytokines including, interferon-gamma (IFN-γ), tumor necrosis factor-alpha (TNF-α), IL-1β, IL-6, IL-8, IL-16, and MIF. (ii) Anti-inflammatory cytokines, IL-4 and IL-10. (iii) Chemokines, CCL group (CCL21, CCL3, CCL27, CCL11, CCL24, CCL26, CCL1, CCL2, CCL8, CCL7, CCL13, CCL22, CCL25, CCL1, CCL19, CCL20, CCL23, and CCL15), CXCL group, CXCL13, CXCL5, CXCL6, CXCL1, CXCL2, CXCL8, IP-10, CXCL11, MIG, CXCL12, and CXCL16, and CX3CL1 group, and iv) growth factors GM-CSF and IL-2.

### Elution protocol from the DBS

Elution buffer was prepared, by adding one protease inhibitor tablet (Roche)/10 ml of phosphate buffer saline (PBS) and refrigerated till further use. DBS samples were taken out of the freezer and kept at room temperature for thirty to forty minutes before elution. Two punches of 6 mm diameter were made in protein saver cards (DBS) from each patient at each time point. After punching one DBS specimen a blank filter paper was punched twice or thrice before punching on the next DBS sample again to prevent any cross-contamination. The cut discs of DBS were placed in a microcentrifuge tube.120 µl of elution buffer was added to each microcentrifuge tube containing the DBS discs and placed on a shaker for 15 min. After taking out of the shaker,  the tubes were kept at room temperature for 1 hour to give time for elution, and were then, centrifuged at 2300×*g* for 10 min. The supernatant from each tube was transferred to another microcentrifuge tube and the elutes were preserved on ice till the start of the experiment.

### Inflammatory biomarkers detection through multiplex microbead immunoassay

The Biorad40 plex Bio-PlexPro™Human Chemokine Panel was used on Luminex® xMAP™ to detect biomarkers from the eluted DBS samples according to the manufacturer's instructions (BioRad, Hercules, CA). Briefly after pre-wetting the plates, 50 μl of 1 × beads were added to the wells and the plates were washed twice with the wash buffer. Afterward, 50 µl of standards, controls, and undiluted eluted samples were added to the respective wells. After one hour's incubation on a shaker at room temperature, plates were washed three times and 25 µl of detection antibodies were added to each well. Following that, incubation was done again for 30 min at room temperature and washed thrice. 50 µl of streptavidin-E was added to the wells. Plates were incubated for another 10 min on a shaker at room temperature and after three steps of washings, resuspended with 125 µl of assay buffer. Plates were read with a Luminex instrument (Luminex 200, Austin Luminex, USA) and data were analyzed using MILLIPLEX Analyst 5.1 software (Merck Millipore Darmstadt, Germany), as per the manufacturer’s instructions.

### Case definition

Cases were defined as (i) TB cases confirmed either by bacteriological confirmation using culture and/or Xpert, or (ii) clinically diagnosed cases/probable TB cases by using a composite reference standard based on signs, symptoms, and laboratory findings consistent with TB and a good response to anti-TB treatment at 2/3 months or/and the end of the treatment. The laboratory findings consistent with TB pleuritis included lymphocytosis, fluid protein level > 3 g/dl, or plasma adenosine deaminase levels > 16 U/L, or concomitant pulmonary TB suggested by positive acid-fast smear and/or chest radiograph. TB lymphadenitis was classified as probable if in addition to the typical clinical signs and symptoms, the histopathology findings were also suggestive of TB lymphadenopathy.

### Assessment of response to treatment

A set of clinical criteria were used as a tool to assess the response to treatment. It included (i) improvement of the presenting symptoms, (ii) regression of the local signs of the disease, such as regression of pleural effusion (estimated by chest ultrasound) and enlarged lymph nodes, and (iii) weight gain. The response was considered good if any two of the three criteria were fulfilled. Based on the response to treatment at the end of 2nd month, the patients were classified into responders (two of the three criteria were fulfilled), and partial responders showing partial clinical response after 2nd month of treatment. Patients were categorized as partial responders by the attending physician if a patient was showing some clinical improvement with weight gain with partial regression of pleural effusion and enlarged lymph nodes. All patients showed a good response at the end of the treatment.

### Statistical methods

Nonparametric statistical analysis was performed by using International Business Machine (IBM)—Statistical Package for Social Sciences (SPSS) Statistics version 26. Wilcoxon test was used to do a paired analysis of levels of biomarkers at 2 and 6 months as compared to the baseline levels. The general significance level was set to 0.05. We used the Bonferroni adjustment for multiple testing, leading to the p-value of 0.025 for 2 tests (0–2 and 0–6). A p-value ≤ 0.025was considered statistically significant. A software library, Python and Data Analysis (Pandas) was used to generate different biosignatures by making combinations of the inflammatory biomarkers which showed statistically significant change with treatment. All possible combinations of the selected biomarkers were computed. The biomarker combinations covering the greatest number of patients and the least number of biomarkers were selected. A ≥ 20% change occurring in any one of the biomarker included in the biosignature was taken as an indication of satisfactory response to treatment in that patient.

### Ethics declarations

The study was approved by the Institutional Review Board, Al. Aleem Medical College & Gulab Devi Educational Complex Lahore (GDEC/18-322) and Regional Committee for Medical and Health Research Ethics, Western-Norway (2018/2392/REK vest). All experiments were performed in accordance with relevant guidelines and regulations. All study participants provided informed consent.

## Supplementary Information

Supplementary Information.

## Data Availability

The datasets generated during and/or analyzed during the current study are available from the corresponding author upon reasonable request.
