# Supplementary material for: Identification of host biomarkers from dried blood spots for monitoring treatment response in extrapulmonary tuberculosis
Source: Sci Rep. 2023 Jan 12;13:599. doi: 10.1038/s41598-022-26823-6 (PMC9837114; doi:10.1038/s41598-022-26823-6)
Supplement: Supplementary file 1 — Supplementary Information. [file 41598_2022_26823_MOESM1_ESM.pdf]

Title

Identification of host biomarkers from dried blood spots for monitoring treatment response in extrapulmonary tuberculosis.

Authors

Shizza Khalid[1*], Atiqa Ambreen[2,3*], Aasia Khaliq[4], Hafeez Ullah[2], Manal Mustafa[5], Tehmina Mustafa[1,6**]

*Shizza Khalid and Atiqa Ambreen share first authorship

Supplementary Data

Supplementary Table 1: List and classification of the biomarkers that were analysed using the Biorad 40 plex Bio-Plex Pro^{TM} Human Chemokine Panel.

<table>
<tr><td>Pro-inflammatory cytokines</td><td colspan="2">Interferon-gamma (IFN-γ), Tumor necrosis factor Alpha (TNF-α), IL-1β, IL-6 and IL-8, IL-16, MIF</td></tr>
<tr><td>Anti-inflammatory cytokines</td><td colspan="2">IL4, IL-10</td></tr>
<tr><td rowspan="3">Chemokines</td><td>CCL</td><td>6Ckine /CCL21, CTACK/CCL27, Eotaxin/CCL11, Eotaxin-2 /CCL24, Eotaxin-3/CCL26, 309 / CCL1, MCP-1/CCL2, MCP-2 /CCL8, MCP-3/CCL7, MCP-4/CCL13, MDC/CCL22, TECK/CCL25, TARC/CCL1, MIP-3β/CCL19, MIP-3α /CCL20, MPIF-1/CCL23, MIP-1δ/CCL15.</td></tr>
<tr><td>CXCL</td><td>BCA-1/CXCL13, ENA-78/CXCL5, GCP-2/CXCL6, Gro-α/CXCL1, Gro-β/CXCL2, IL-8/CXCL8, IP-10/CXCL10, I-TAC/CXCL11, MIG/CXCL9, SDF-1α+β/CXCL12, SCYB16/CXCL16.</td></tr>
<tr><td>CX3CL</td><td>Fractalkine/CX3CL1</td></tr>
<tr><td>Growth factors</td><td colspan="2">Granulocyte-macrophage colony-stimulating factor (GM-CSF), IL-2</td></tr>
</table>

Supplementary Figure 1.

Box plots showing levels of inflammatory biomarkers in dried blood spots in TB pleuritis patients at baseline (0M), 2nd month (2M) and 6th month(6M) of treatment. The levels at 2M and 6M were compared with the baseline values. A p-value < 0.05 was considered significant. The boxes represent the median and interquartile range, while the whisker represents the minimum and maximum values. Different time points of treatment are shown on x axis. n; number of patients

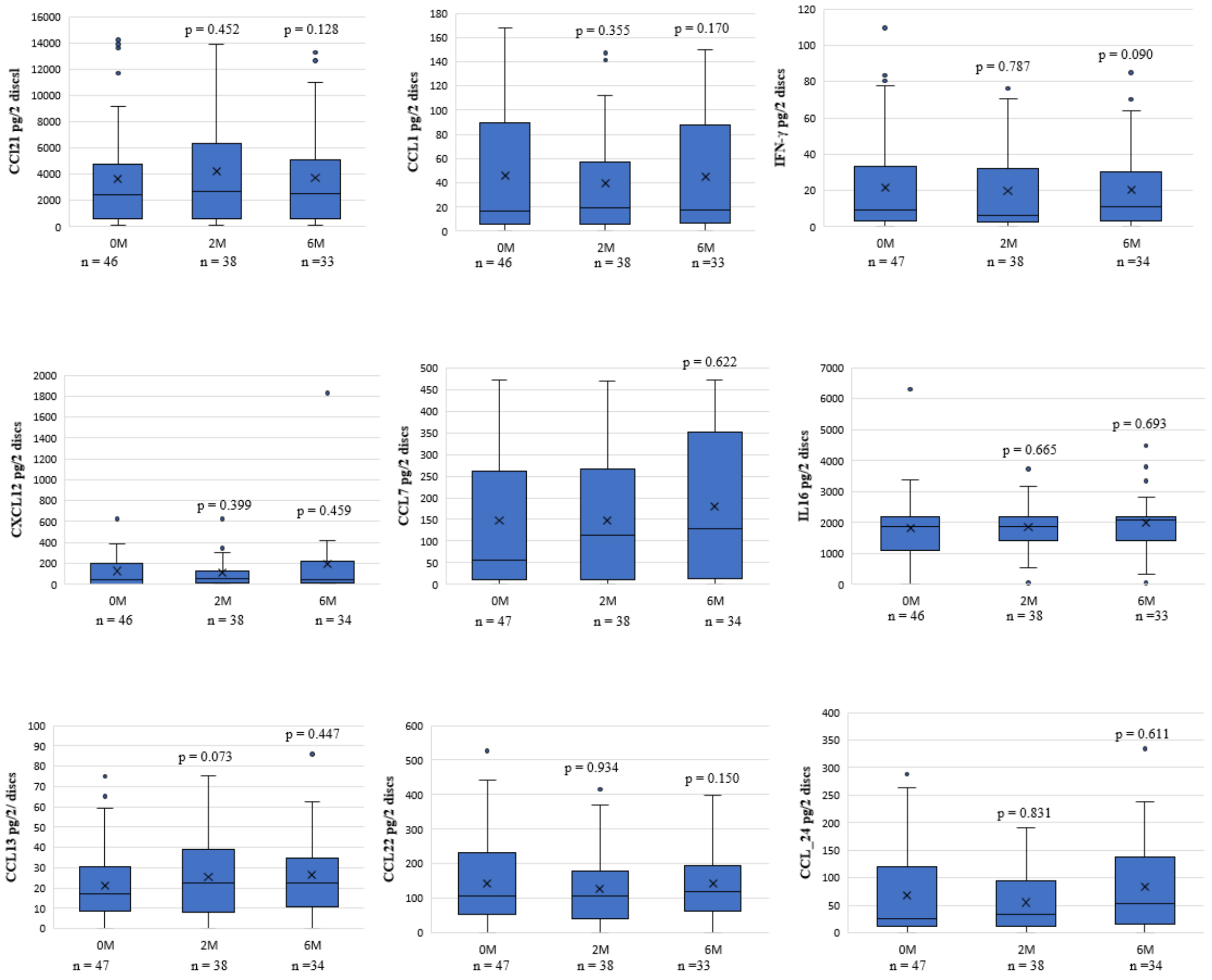

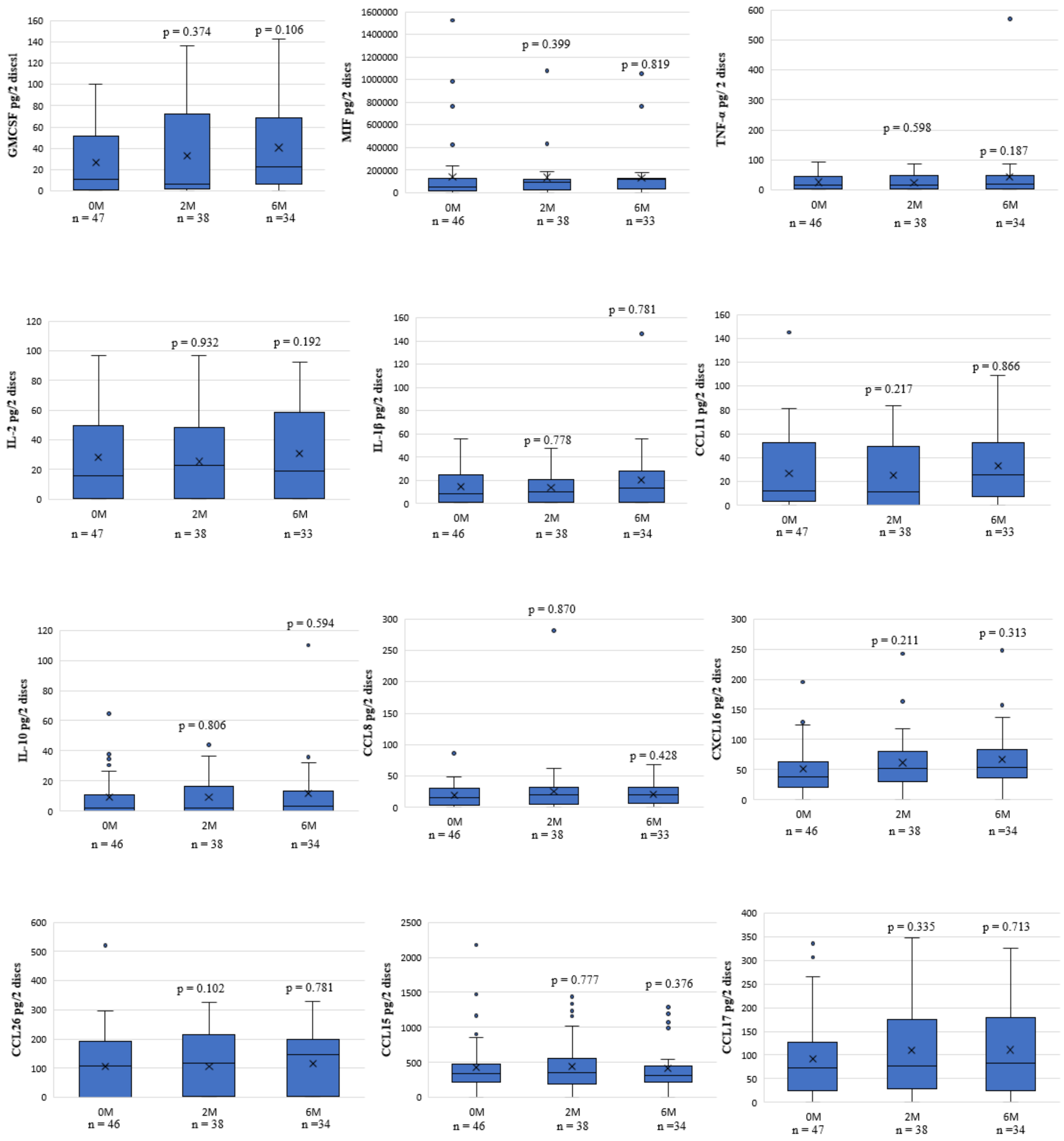
GMCSF pg/2 discs1
p = 0.374
p = 0.106
0M n = 47
2M n = 38
6M n =34
MIF pg/2 discs
p = 0.399
p = 0.819
0M n = 46
2M n = 38
6M n =33
TNF-α pg/2 discs
p = 0.598
p = 0.187
0M n = 46
2M n = 38
6M n =34
IL-2 pg/2 discs
p = 0.932
p = 0.192
0M n = 47
2M n = 38
6M n =33
IL-1β pg/2 discs
p = 0.781
p = 0.778
0M n = 46
2M n = 38
6M n =34
CCL11 pg/2 discs
p = 0.217
p = 0.866
0M n = 47
2M n = 38
6M n =33
IL-10 pg/2 discs
p = 0.594
p = 0.806
0M n = 46
2M n = 38
6M n =34
CCL8 pg/2 discs
p = 0.870
p = 0.428
0M n = 46
2M n = 38
6M n =33
CXCL16 pg/2 discs
p = 0.211
p = 0.313
0M n = 46
2M n = 38
6M n =34
CCL26 pg/2 discs
p = 0.102
p = 0.781
0M n = 46
2M n = 38
6M n =34
CCL15 pg/2 discs
p = 0.777
p = 0.376
0M n = 46
2M n = 38
6M n =34
CCL17 pg/2 discs
p = 0.335
p = 0.713
0M n = 47
2M n = 38
6M n =34

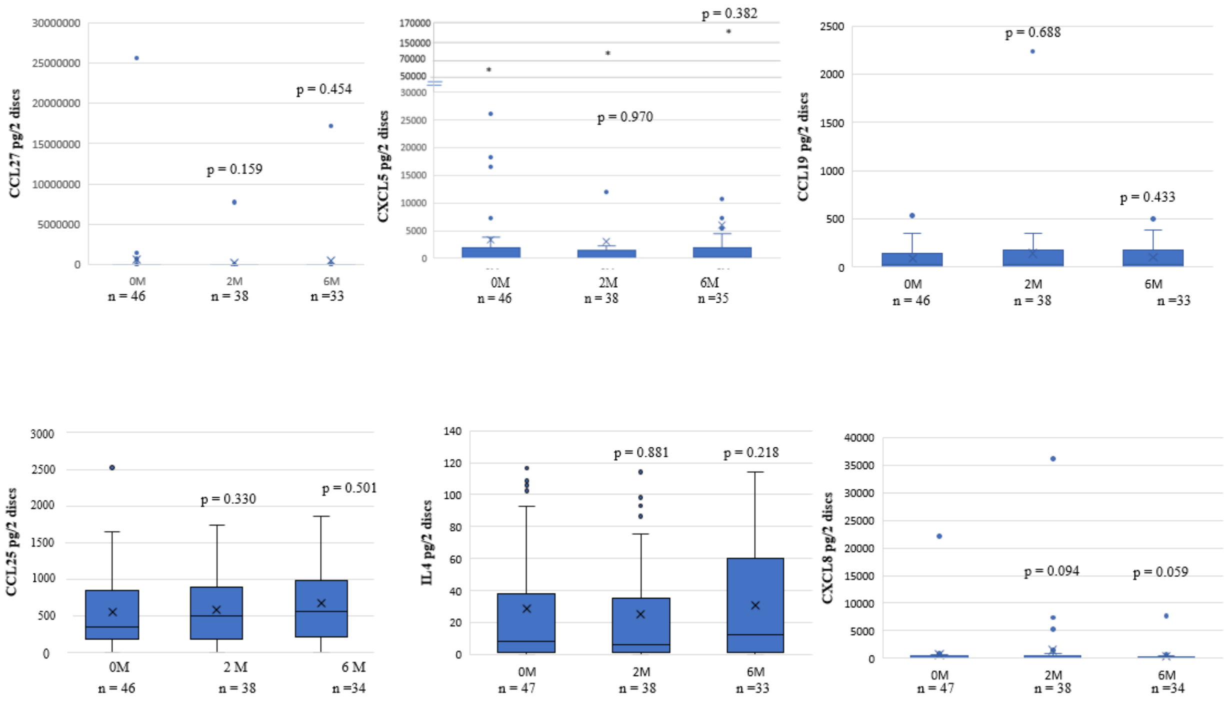
CCL27 pg/2 discs
30000000
25000000
20000000
15000000
10000000
5000000
0
p = 0.159
p = 0.454
0M
2M
6M
n = 46
n = 38
n =33
CXCL5 pg/2 discs
170000
150000
70000
50000
30000
25000
20000
15000
10000
5000
0
p = 0.382
p = 0.970
0M
2M
6M
n = 46
n = 38
n =35
CCL19 pg/2 discs
2500
2000
1500
1000
500
0
p = 0.688
p = 0.433
0M
2M
6M
n = 46
n = 38
n =33
CCL25 pg/2 discs
3000
2500
2000
1500
1000
500
0
p = 0.330
p = 0.501
0M
2 M
6 M
n = 46
n = 38
n =34
IL4 pg/2 discs
140
120
100
80
60
40
20
0
p = 0.881
p = 0.218
0M
2M
6M
n = 47
n = 38
n =33
CXCL8 pg/2 discs
40000
35000
30000
25000
20000
15000
10000
5000
0
p = 0.094
p = 0.059
0M
2M
6M
n = 47
n = 38
n =34

Supplementary Figure 2. Box plots showing levels of inflammatory biomarkers in dried blood spots in TB lymphadenitis patients at baseline (0M), 2nd month (2M) and 6th month(6M) of treatment. The levels at 2M and 6M were compared with the baseline values. A p-value < 0.05 was considered significant. The boxes represent the median and interquartile range, while the whisker represents the minimum and maximum values. Different time points of treatment are shown on x axis. n; number of patients

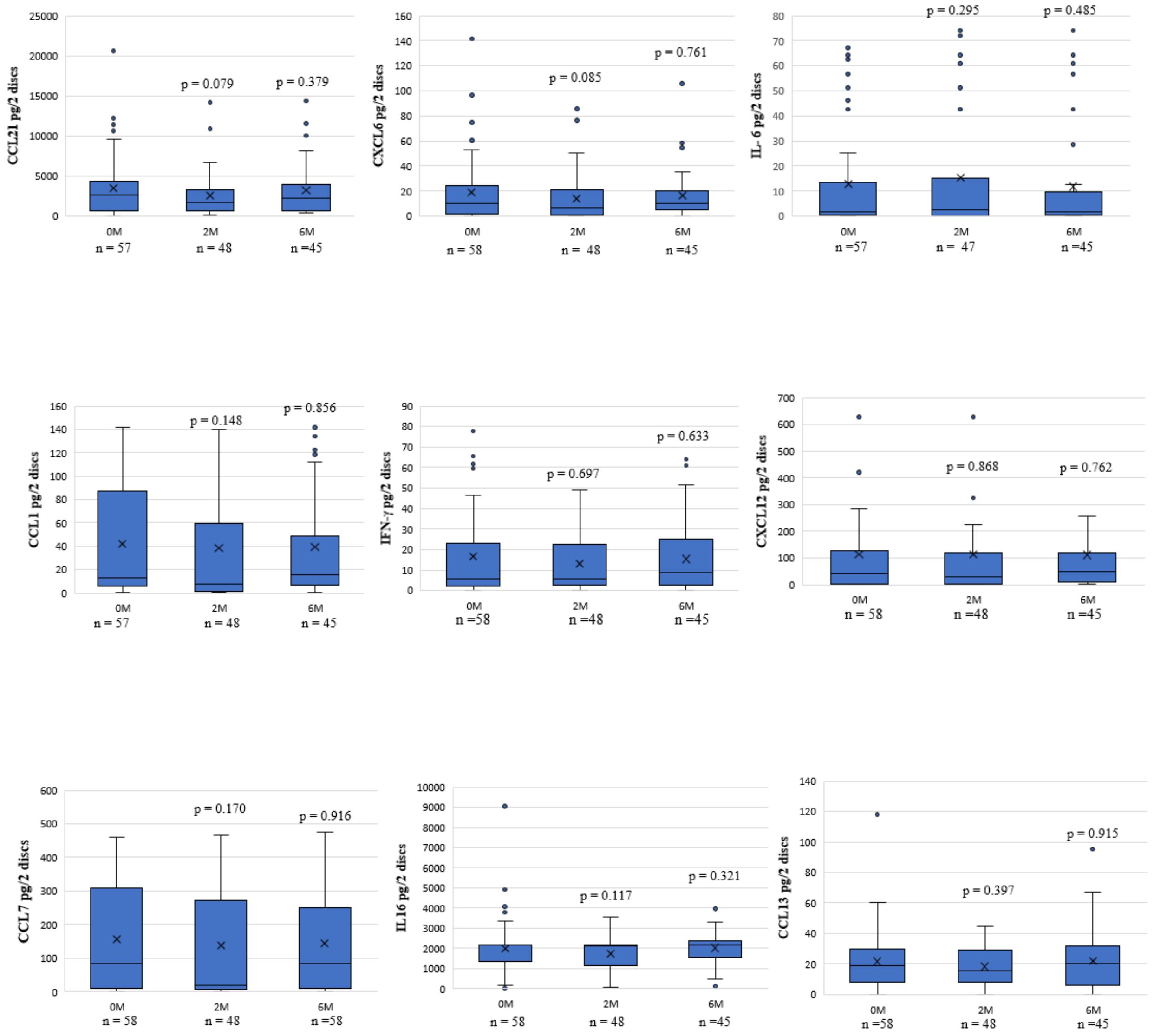

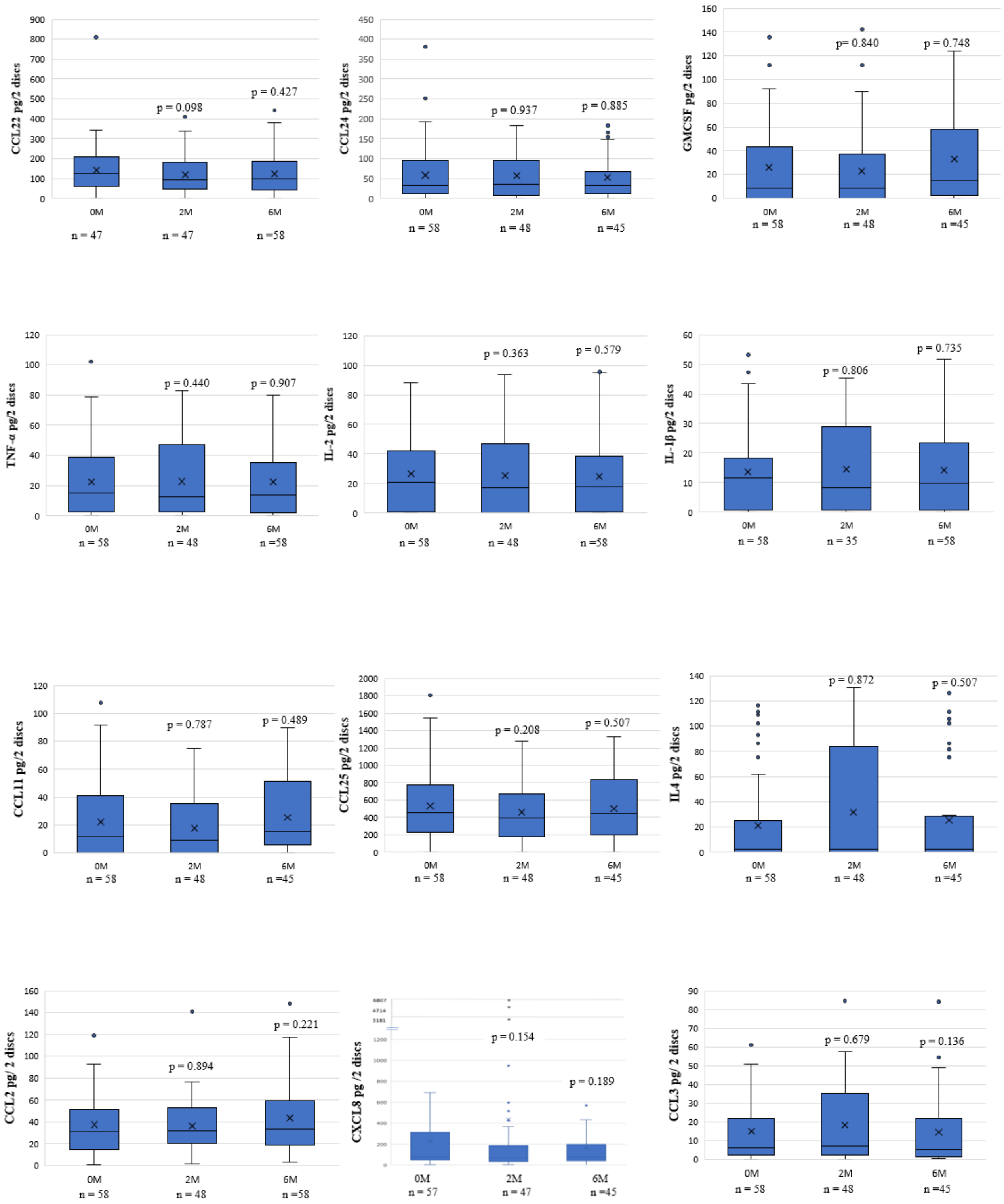
CCL22 pg/2 discs
p = 0.098
p = 0.427
0M
2M
6M
n = 47
n = 47
n =58
CCL24 pg/2 discs
p = 0.937
p = 0.885
n = 58
n = 48
n =45
GMCSF pg/2 discs
p= 0.840
p = 0.748
n = 58
n = 48
n =45
TNF-α pg/2 discs
p = 0.440
p = 0.907
n = 58
n = 48
n =58
IL-2 pg/2 discs
p = 0.363
p = 0.579
n = 58
n = 48
n =58
IL-1β pg/2 discs
p = 0.806
p = 0.735
n = 58
n = 35
n =58
CCL11 pg/2 discs
p = 0.787
p = 0.489
n = 58
n = 48
n =45
CCL25 pg/2 discs
p = 0.208
p = 0.507
n = 58
n = 48
n =45
IL4 pg/2 discs
p = 0.872
p = 0.507
n = 58
n = 48
n =45
CCL2 pg/ 2 discs
p = 0.894
p = 0.221
n = 58
n = 48
n =58
CXCL8 pg /2 discs
p = 0.154
p = 0.189
n = 57
n = 47
n =45
CCL3 pg/ 2 discs
p = 0.679
p = 0.136
n = 58
n = 48
n =45

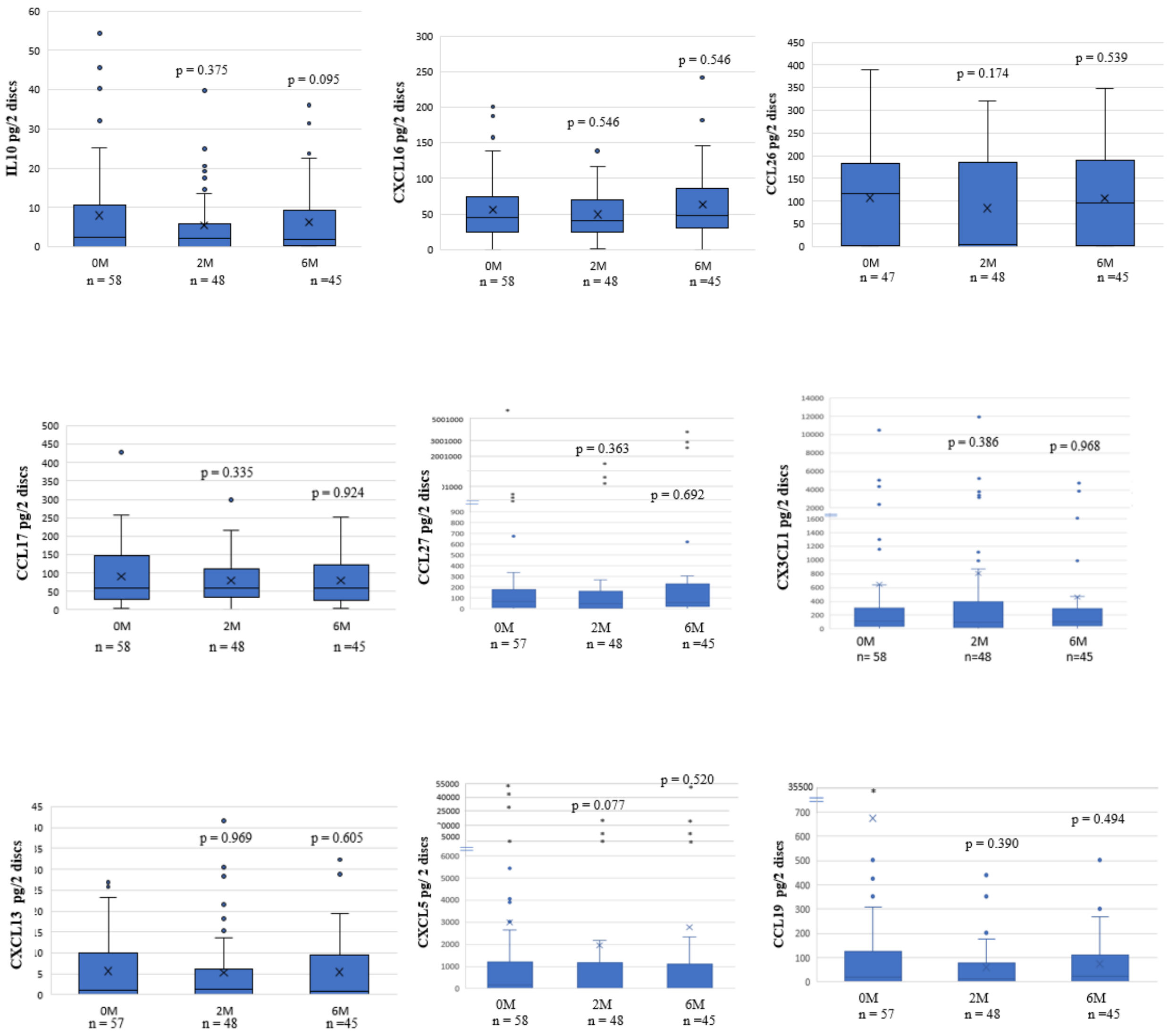

IL10 pg/2 discs
p = 0.375
p = 0.095
0M n = 58
2M n = 48
6M n =45
CXCL16 pg/2 discs
p = 0.546
p = 0.546
0M n = 58
2M n = 48
6M n =45
CCL26 pg/2 discs
p = 0.174
p = 0.539
0M n = 47
2M n = 48
6M n =45
CCL17 pg/2 discs
p = 0.335
p = 0.924
0M n = 58
2M n = 48
6M n =45
CCL27 pg/2 discs
p = 0.363
p = 0.692
0M n = 57
2M n = 48
6M n =45
CX3CL1 pg/2 discs
p = 0.386
p = 0.968
0M n= 58
2M n=48
6M n=45
CXCL13 pg/2 discs
p = 0.969
p = 0.605
0M n = 57
2M n = 48
6M n =45
CXCL5 pg/2 discs
p = 0.077
p = 0.520
0M n = 58
2M n = 48
6M n =45
CCL19 pg/2 discs
p = 0.390
p = 0.494
0M n = 57
2M n = 48
6M n =45
